# Supplementary material for: Comparative multiomics analysis of cell physiological state after culture in a basket bioreactor
Source: Sci Rep. 2022 Nov 23;12:20161. doi: 10.1038/s41598-022-24687-4 (PMC9686226; doi:10.1038/s41598-022-24687-4)
Supplement: Supplementary file 1 — Supplementary Information 1. [file 41598_2022_24687_MOESM1_ESM.zip › raw data/Transcriptomic raw data/6.Enrichment/2.KEGG/TE1vsTC1/GSEA/VIRAL_CARCINOGENESIS(CSAB05203).html]

Details for gene set VIRAL\_CARCINOGENESIS(CSAB05203)[GSEA]

|  || Dataset | readcount.sample |
| Phenotype | sample.cls |
| Upregulated in class | TE1 |
| GeneSet | VIRAL\_CARCINOGENESIS(CSAB05203) |
| Enrichment Score (ES) | 0.53009486 |
| Normalized Enrichment Score (NES) | 1.1071396 |
| Nominal p-value | 0.19689737 |
| FDR q-value | 0.97292686 |
| FWER p-Value | 1.0 |
Table: GSEA Results Summary

  

Fig 1: Enrichment plot: VIRAL\_CARCINOGENESIS(CSAB05203)      
 Profile of the Running ES Score & Positions of GeneSet Members on the Rank Ordered List

  

| PROBE | DESCRIPTION (from dataset) | GENE SYMBOL | GENE\_TITLE | RANK IN GENE LIST | RANK METRIC SCORE | RUNNING ES | CORE ENRICHMENT || 1 | novel.575 | - |  |  | 1918 | 2.133 | 0.2572 | Yes |
| 2 | novel.578 | - |  |  | 2496 | 1.858 | 0.5301 | Yes |
| 3 | novel.538 | - |  |  | 11740 | 0.910 | 0.2637 | No |
| 4 | novel.497 | - |  |  | 13488 | 0.801 | 0.3145 | No |
| 5 | novel.361 | - |  |  | 17647 | 0.519 | 0.2123 | No |
Table: GSEA details [plain text format]

  

Fig 2: VIRAL\_CARCINOGENESIS(CSAB05203)      
 Blue-Pink O' Gram in the Space of the Analyzed GeneSet

  

Fig 3: VIRAL\_CARCINOGENESIS(CSAB05203): Random ES distribution      
 Gene set null distribution of ES for **VIRAL\_CARCINOGENESIS(CSAB05203)**
